# Mapping Shunting Paths at the Surface of Cu_2_ZnSn(S,Se)_4_ Films via Energy-Filtered Photoemission Microscopy

**DOI:** 10.1016/j.isci.2018.10.004

**Published:** 2018-10-13

**Authors:** Devendra Tiwari, Mattia Cattelan, Robert L. Harniman, Andrei Sarua, Ali Abbas, Jake W. Bowers, Neil A. Fox, David J. Fermin

**Affiliations:** 1 School of Chemistry, University of Bristol, Bristol BS8 1TS, UK; 2 H H Wills Physics Laboratory, University of Bristol, Bristol BS8 1TL, UK; 3 Centre for Renewable Energy Systems Technology (CREST), Wolfson School of Mechanical, Electrical and Manufacturing Engineering, Loughborough University, Loughborough LE11 3TU, UK

**Keywords:** Chemistry, Materials Science, Energy Materials

## Abstract

The performance of Cu_2_ZnSn(S,Se)_4_ thin-film solar cells, commonly referred to as kesterite or CZTSSe, is limited by open-circuit voltage (V_OC_) values less than 60% of the maximum theoretical limit. In the present study, we employ energy-filtered photoemission microscopy to visualize nanoscale shunting paths in solution-processed CZTSSe films, which limit the V_OC_ of cells to approximately 400 mV. These studies unveil areas of local effective work function (LEWF) narrowly distributed around 4.9 eV, whereas other portions show hotspots with LEWF as low as 4.2 eV. Localized valence band spectra and density functional theory calculations allow rationalizing the LEWF maps in terms of the CZTSSe effective work function broadened by potential energy fluctuations and nanoscale Sn(S,Se) phases.

## Highlights

• Exploring Cu_2_ZnSn(S,Se)_4_ films with energy-filtered photoelectron emission microscopy
• Sub-micrometer hotspots of low effective work function are revealed
• Valence band spectra of hotspots suggests discrete Sn(S,Se) surface domains
• Sn(S,Se) domains may act as carrier shunts in PV devices

## Graphical Abstract

**Figure:**
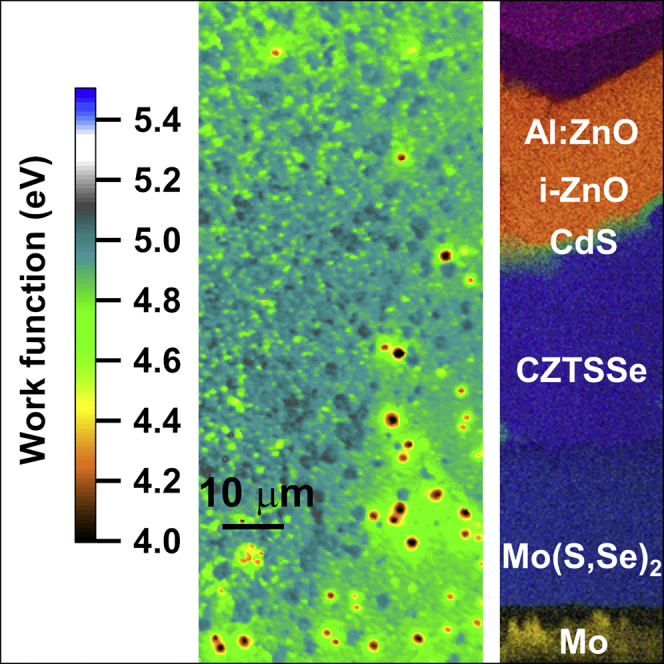

## Introduction

Thin-film photovoltaic (PV) solar cells comprise approximately 10% of the PV installed capacity worldwide (Fraunhofer ISE, 2017, Haegel et al., 2017). Considering the exponential increase in the PV market over the last 10 years, it is crucial to develop new scalable technologies based on low-cost earth-abundant materials (Steinmann et al., 2015, Fraunhofer ISE, 2017). Cu_2_ZnSn(S,Se)_4_ (CZTSSe) largely fulfills the key requirements in terms of optical properties, electronic structure, Earth abundance, and stability, although device power conversion efficiency (η) has been limited by low open-circuit voltage (*V*_OC_) and fill factor (*FF*) (Polizzotti et al., 2013, Kumar et al., 2015, Liu et al., 2016, Wallace et al., 2017). The most efficient cells certified to date, with a band gap of 1.1 eV, have shown *V*_OC_ values of 513 mV (η = 12.6%) (Wang et al., 2014), 521 mV (η = 12.3%) (Yang et al., 2016), and 670 mV (11.9%) (Antunez et al., 2017b). These cells have been fabricated by physical vapor deposition (Yang et al., 2016) as well as solution-based methods (Wang et al., 2014), suggesting that key power conversion losses are linked to intrinsic material properties rather than the preparation method.

Low *V*_OC_ values have been linked to non-optimal band alignment at CZTSSe/CdS boundary as well as elemental disorder in CZTSSe, mainly point defects such as Cu_Zn_ antisites (Siebentritt, 2013, Bourdais et al., 2016). Clustering of these point defects into domains can lead to potential energy fluctuations, which manifest themselves as band tails. Broadening and complex temperature dependence of photoluminescence responses provide the clearest manifestation of band tails in these materials (Tiwari et al., 2017b). Elemental disorder has also been investigated by X-ray and neutron diffraction (Schorr, 2011, Tobbens et al., 2016), as well as solid-state nuclear magnetic resonance (Paris et al., 2015). On the other hand, density functional theory (DFT) analysis also predicts that Sn disorder may generate states deeper in the band gap, which may act as recombination centers (Chen et al., 2009). Recently, we have shown the presence of Sn antisite domain boundaries in CZTS nanostructures employing atomic-resolution transmission electron microscopy (Kattan et al., 2016). Nanoscale domains of secondary phases have also been observed employing atom-probe tomography (Tajima et al., 2014) and high-resolution cathodoluminescence (Mendis et al., 2018). Systematic approaches to reduce bulk composition disorder via controlled annealing and the introduction of additives (e.g., alkali metal, Ag, Cd, Ge, and Sb) have led to improvement in device efficiency, yet *V*_OC_ values remain in the 500–600 mV range (Johnson et al., 2014, Kumar et al., 2015, Su et al., 2015, Tiwari et al., 2016, Qi et al., 2017, Giraldo et al., 2018, Sai Gautam et al., 2018). These observations have focused our attention away from bulk and into interfacial defects as the key factor determining voltage losses, which is emphasized by the fact that little is known about the surface structure of these complex materials.

Experimental evidences have shown the importance of understanding the structure of the buried junctions involving the absorber layer, namely, the Mo/CZTSSe and the CZTSSe/CdS interfaces. For instance, Haight and co-workers exfoliated CZTSSe cells from the Mo back-contact and mechanically reconnected them achieving a substantial increase in power conversion efficiency (Antunez et al., 2017a). This is due to the partial selenization of the Mo film during the thermal annealing step. However, investigating the CZTSSe/CdS boundary with the penetration depth offered by photoemission spectroscopy is significantly challenging (Bär et al., 2017).

In this work, we employ for the first time sub-micron-resolution energy-filtered photoemission microscopy (EF-PEEM) to examine the complex and poorly understood surface electronic structure of CZTSSe thin films. Our investigation focuses on thin films generated by annealing of molecular precursors, which are characterized by a high degree of crystallinity and phase purity and power conversion efficiencies of 5.7% under AM 1.5G illumination (Tiwari et al., 2017b). Photoemission maps show a distribution in the onset energy of secondary electron emission, which we rationalized in terms of local effective work functions (LEWF). Analysis of the valence band spectra, supported by DFT supercell calculations, provides a strong link between the most prominent LEWF features and the main CZTSSe phase. The photoemission landscape also unveils sub-micron domains of low LEWF, acting as shunting path for photogenerated electrons. Local valence band spectrum reveals that these photoemission hotspots correspond to Sn(S,Se) surface domains. We conclude that these surface domains, not detectable by conventional spectroscopic and diffraction techniques, can play a substantial role in the voltage losses in devices.

## Results

### Solution-Based CZTSSe: Device Performance

**Figure 1:**
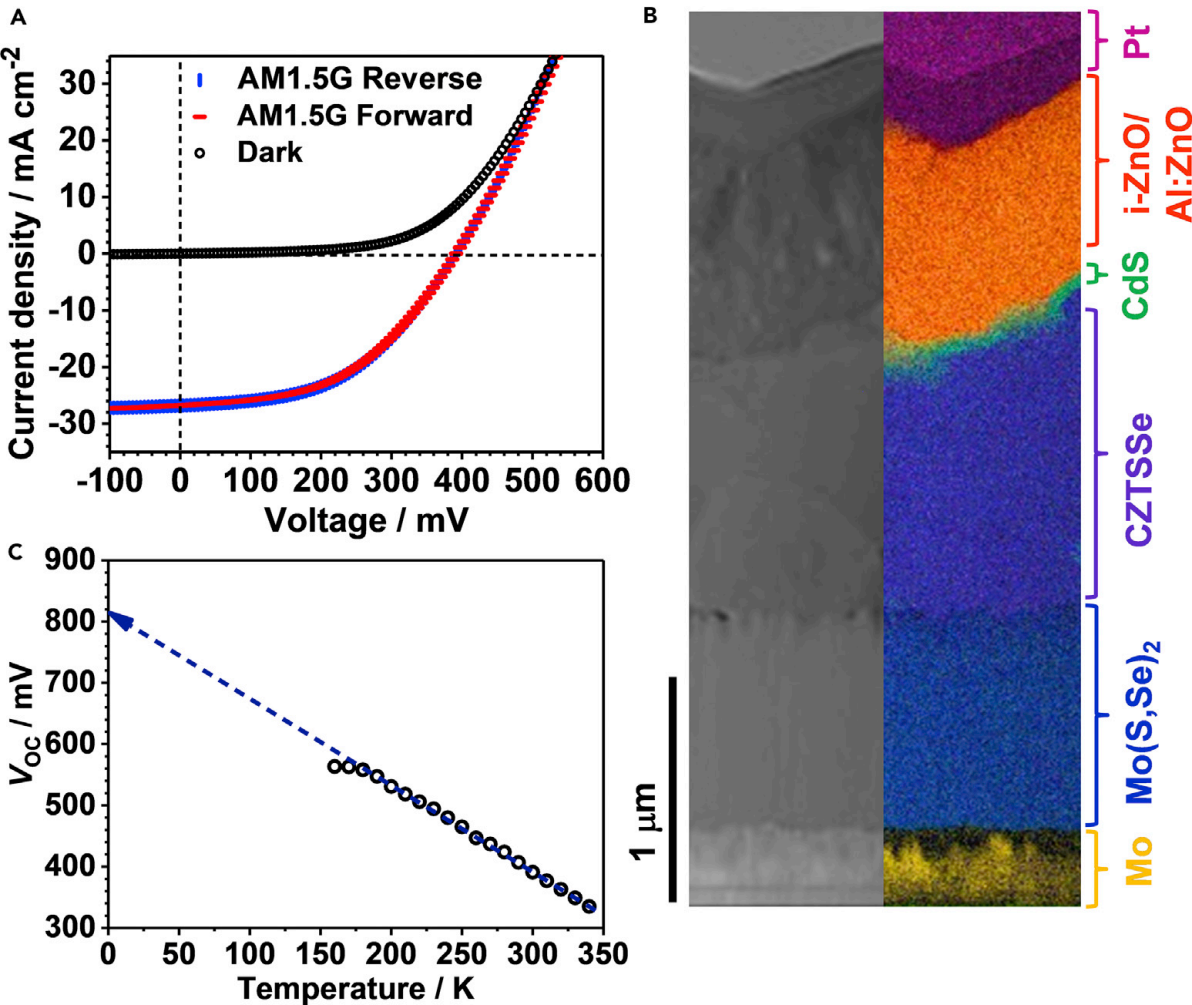
Characteristic CZTSSe Device Performance (A) *J-V* curve of a CZTSSe solar cell (total area: 0.25 cm^2^) in dark and under AM1.5G illumination, recorded upon scanning the voltage toward forward and reverse bias. The cell shows an open-circuit voltage (*V*_OC_), short-circuit density (*J*_SC_), and fill factor (*FF*) of 391 mV, 26.8 mA cm^2^, and 48.3%, respectively. (B) High-angle annular dark-field imaging-scanning transmission electron microscopic image and elemental mapping of a device cross section generated by focused ion beam, with colors identifying key elements: Mo (yellow), Cu (purple), Cd (green), Zn (orange), and Pt (violet). Mo(S,Se)_2_ layer (blue) is obtained by combining the contributions of Mo, S, and Se. (C) Temperature dependence of open-circuit voltage for calculating the activation energy of dominant recombination pathway.

Figure 1A shows a characteristic J-V curve of a CZTSSe device (0.25 cm^2^) under AM1.5G illumination at room temperature. The CZTSSe film was obtained by annealing of a single molecular precursor spin-coated onto Mo-coated glass in the presence of Se, based on a protocol reported elsewhere (Tiwari et al., 2016, Tiwari et al., 2017b). The film is etched in KCN before chemical bath deposition of CdS and deposition of i-ZnO and Al-doped ZnO via radio frequency (RF) sputtering. Figure 1B shows a cross-sectional scanning transmission electron micrograph of a section of a typical device along with the elemental map by energy-dispersive analysis of X-rays (EDX). The color code in the EDX cross section is associated with the key contrasting element in each layer, i.e., Mo (yellow), Cu (purple), Cd (green), Zn (orange), and Pt (violet), with the latter deposited as part of focused ion beam preparation. The Mo(S,Se)_2_ layer (blue) is obtained by combining the contributions from Mo, S, and Se. The CZTSSe film thickness is approximately 1.3 μm, with the CdS and TCO layers growing highly conformally over the absorber film. The highest conversion efficiency is 5.7%, and the dispersion of the key cell metrics is below 15% for over 20 cells (Tiwari et al., 2017b). Neither top metal contact nor antireflective coating was used in cell fabrication. Fitting the forward J-V curve in the dark to the diode equation including shunt and series resistance terms yielded an ideality factor close to 2, indicating thermally activated recombination channels. The low *FF* is mainly linked to partial selenization of the Mo surface, also seen in Figure 1B, which generates back-contact barriers with heights in the range of 300 meV (Tiwari et al., 2017b).

The key factor limiting the power conversion efficiency of these cells is the *V*_OC_. Figure 1C shows the temperature dependence of *V*_OC_, which can be described in terms of (Hages et al., 2014)

(Equation 1) $$\text{e}V_{\text{OC}}\;=\;E_{A,Voc}\;\text{-}\text{n}{\text{k}}_{B}T\left(\frac{J_{00}}{J_{\text{SC}}}\right)$$

where *J*_00_ is the reverse saturation current density, *n* is the ideality factor, and *E*_A,Voc_ is the activation energy for recombination. Neglecting the temperature dependence of *J*_00_, the extrapolation of the linear portion of the graph of *T* versus *V*_OC_ results in *E*_A,Voc_ = 0.82 eV. This value is lower than the film band gap, *E*_g_ = 1.18 eV, estimated from diffuse reflectance measurements and the device external quantum efficiency (EQE) spectra. *E*_A,Voc_ values of 1.00 eV (η = 10.1%)^21^ and 0.97 eV (η = 9.66%) (Barkhouse et al., 2012) have been reported, showing a correlation between this parameter and the power conversion efficiency.

### CZTSSe Thin Film: Topography and Energy-Filtered Photoemission Microscopy

**Figure 2:**
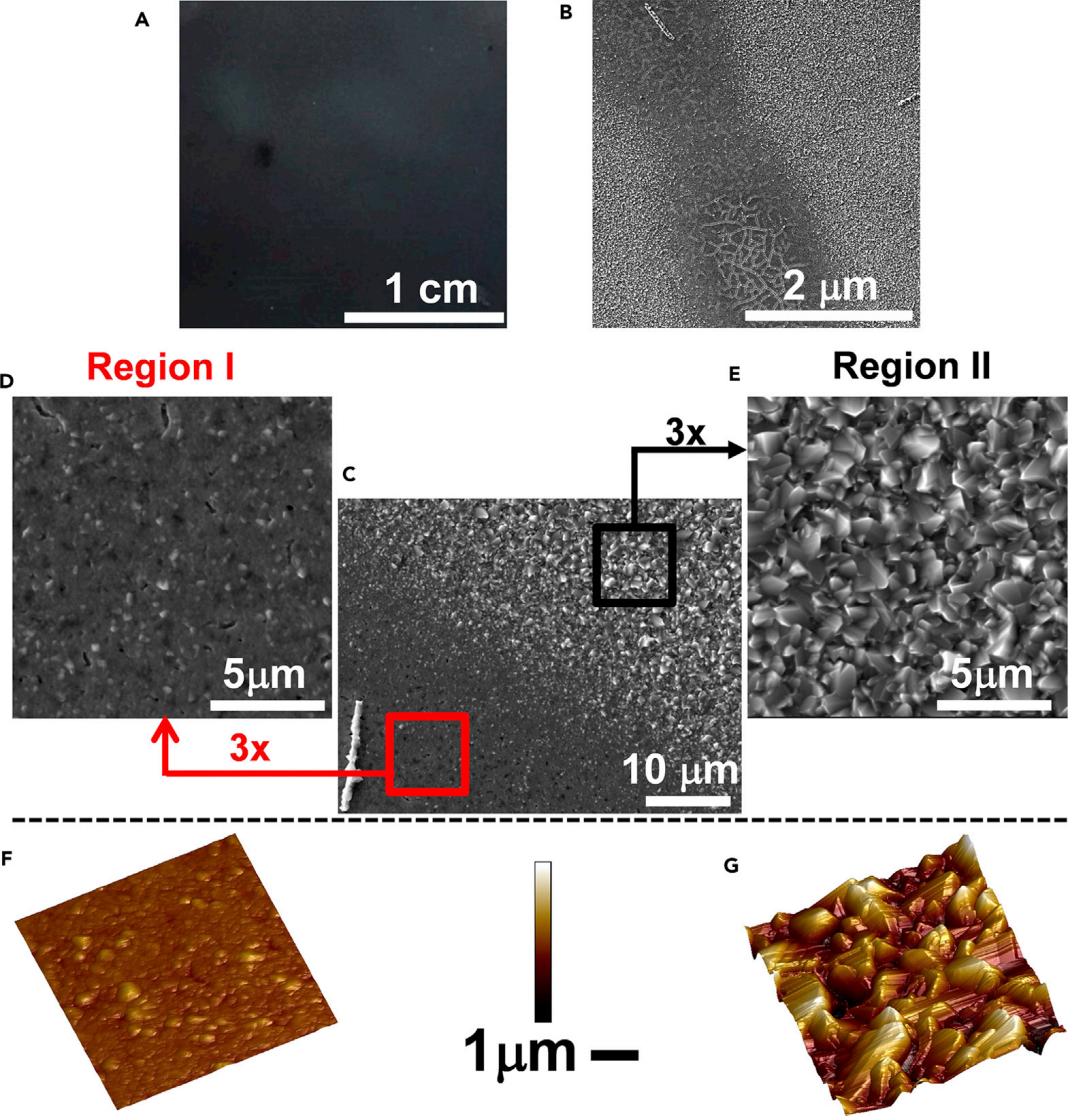
Topographic Features of CZTSSe Films (A–G) (A) Macroscopic photograph of CZTSSe film processed on 2.5 × 2.5 cm^2^-Mo-coated substrate; (B) low-magnification scanning electron micrograph of the film showing “dark” (region I) and “bright” (region II) areas; (C) scanning electron micrograph of a boundary between regions I and II; (D and E) high-resolution scanning electron micrographs of regions I and II; (F and G) topographic atomic force microscopic images of regions I and II.

The topography of freshly etched CZTSSe films imaged over several length scales is illustrated in Figure 2. These films are prepared in a manner similar to those used for device fabrication. The macroscopic image (Figure 2A) shows very little contrast over regions in the centimeter length scale, whereas the scanning electron micrograph in Figure 2B reveals more contrast at the micrometer scale. The regions of darker contrast in the scanning electron micrograph can be observed distributed across the surface, with the brighter regions being the most prominent. Figure 2C zooms in the boundary between the two regions, showing clearly continuity without any cracks. The differences in topography can be clearly seen on the higher resolution scanning electron and atomic force micrographs in Figures 2D–2G. The area with flatter topography (Figure 2F) exhibits a roughness of 32 nm (root mean square [RMS] across 5 × 5 μm^2^ areas, whereas Figure 2E shows larger grains with a roughness of 148 nm. For the rest of the discussion, we shall be referring to these regions as I and II, respectively. The corresponding conductance maps (Supplemental Information, Figure S1) show that the topographically smoother area (region I) is characterized by an rms conductance of 2.9 pA, whereas the rougher region II is characterized by an rms value of 38 pA. Although a contrast in topography and conductance can be observed in these domains, differences are relatively small at the macroscopic level, demonstrating the high quality of the thin-film materials obtained by our solution-based deposition method.

To rationalize the electronic structure of the CZTSSe surface, EF-PEEM studies were performed employing a NanoESCA II (Scienta Omicron). The EF-PEEM analyzer integrates an aberration-compensated energy filter, providing high resolution in real space and energy. Surface preparation is of paramount importance to reproduce these studies. The CZTSSe sample was freshly etched in KCN and examined by X-ray photoelectron spectroscopy (XPS) to determine the surface composition. As shown in the Supplemental Information (Figure S2 and Table S1), Ar plasma etching was optimized to avoid changes in the surface composition. Indeed, a dose of 0.5 kV for 300 s substantially reduced carbon and oxygen impurities, whereas doses in the range of 1 or 1.5 kV and times exceeding 7 min showed substantial changes in metal-cation ratio. It was also observed that the overall surface composition is Zn rich, in agreement with previous studies by Repins and co-workers (Bar et al., 2011). After surface preparation and XPS analysis, the sample was transferred under ultra-high vacuum conditions to the EF-PEEM chamber.

**Figure 3:**
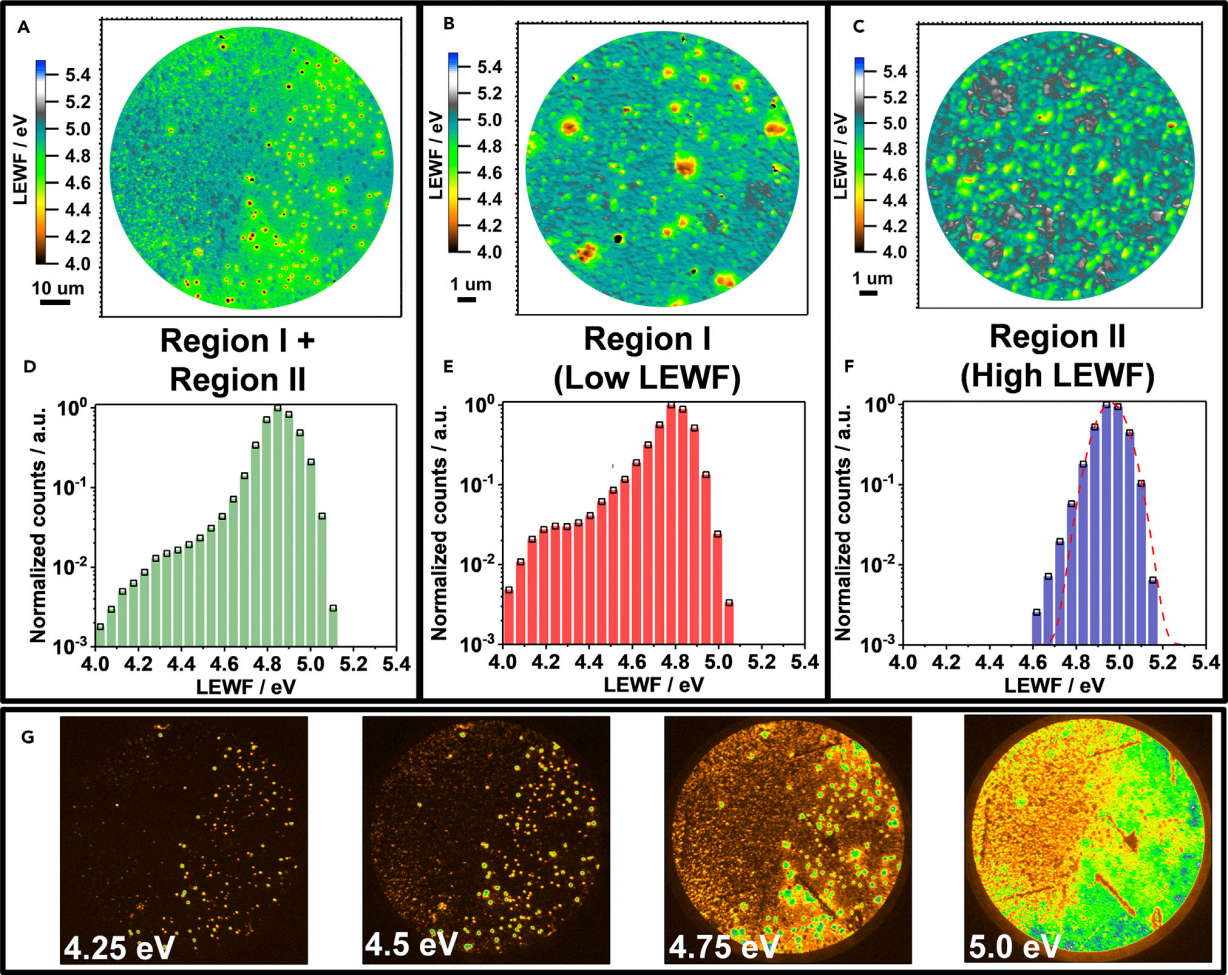
Local Effective Work Function Maps of CZTSSe Films (A–G) (A) LEWF maps of CZTSSe films: broad area scan (FoV 93.2 μm) displaying the boundary between two regions with different LEWF landscape; high-resolution scans (FoV 14.6 μm) of (B) region I and (C) region II; histograms associated with the LEWF maps with (D) large and (E and F) small FoV; (G) photoemission maps (FoV 93.2 μm) recorded at different *E*−*E*_f_ values. A video of the photoemission maps showing electrons emitted with different kinetic energies across the surface is provided in Video S1.

**Figure 4:**
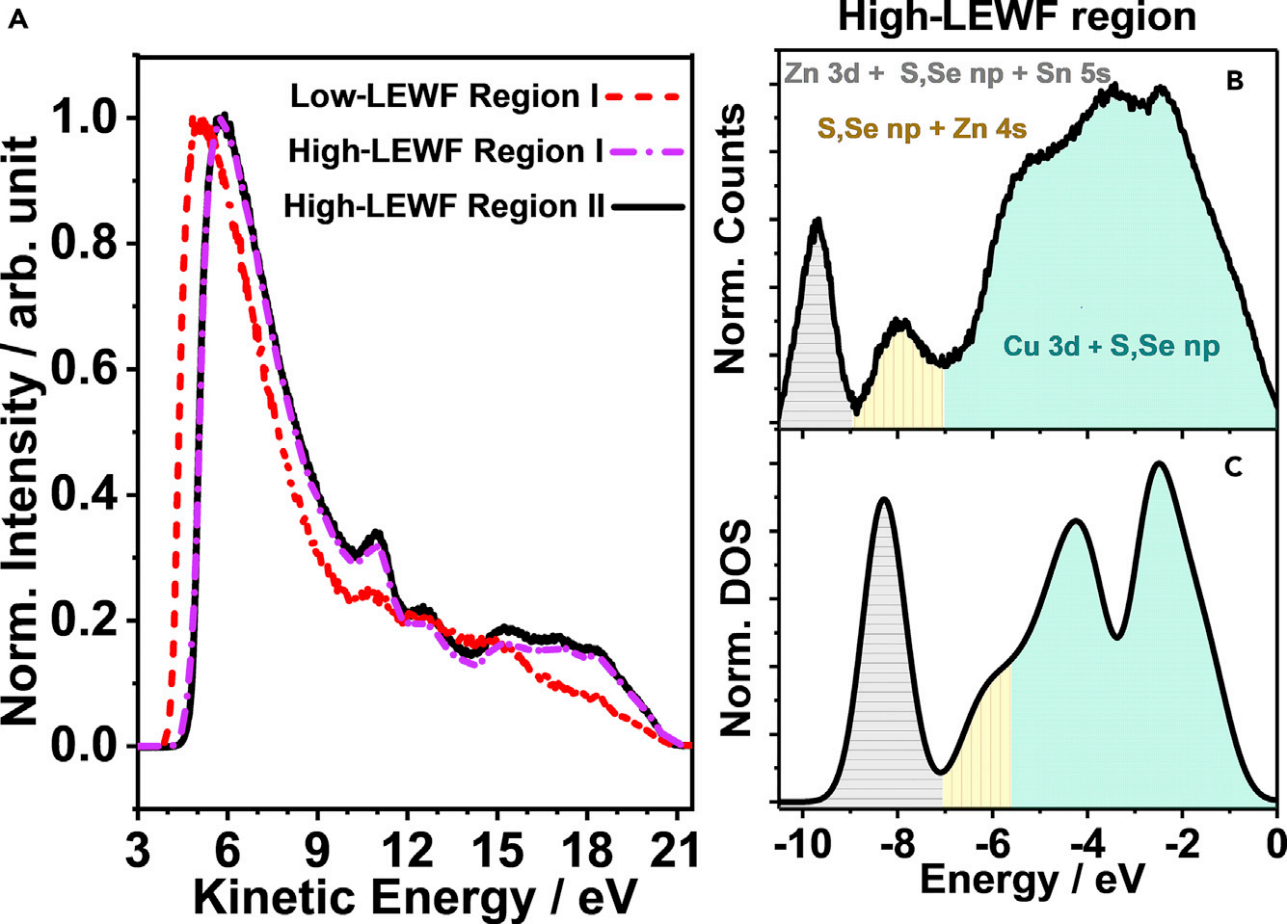
Localized Valence Band Spectra of the CZTSSe Films (A) Local photoemission spectra recorded in areas of high- and low-LEWF values. The LEWF is extracted from the onset of the secondary photoemission edge after fitting an error function. (B) Valence band spectra of high-LEWF domain at the surface after subtracting the incident photon energy (21.2 eV) and applying a Tougaard background correction. (C) Calculated DOS of the valence band of Cu_2_ZnSn(S_0.25_Se_0.75_)_4_ DOS employing HSE06 functional and plotted with 0.4-eV Gaussian smearing to match the broadening in the experimental measurements. Elemental contributions to the valence and conduction band DOS are shown in Figure S6.

Figure 3 displays spatially resolved maps of the electron emission threshold of secondary electrons from a clean CZTSSe surface under He I (21.2 eV) excitation. Photoemission spectra exhibit high and low cutoff values, which are linked to the valence band edge and the emission threshold of secondary electrons, respectively (see, for instance, Figure 4A) (Cahen and Kahn, 2003). In principle, the NanoESCA II detector allows estimating the work function (WF) directly from the low energy emission threshold (Escher et al., 2005). However, this parameter is also dependent on a number of variables including surface potential barriers, local electrostatic fields, and topography. Consequently, we will define the secondary electron energy threshold as LEWF.

Figure 3A displays a very complex photoemission landscape across a 93.2-μm field of view (FoV), including portions of regions I and II (Figure 2). The map shows a broad range of values, with scattered spots exhibiting LEWF as low as 4.2 eV. Higher resolution maps of regions I and II (FoV 14.6 μm) are displayed in Figures 3B and 3C. The topographically smoother region I is characterized by domains of low LEWF (red spots) in the range of 200 nm to a few micrometers, whereas region II shows a much narrower distribution. This is clearly seen in the corresponding histograms in Figures 3D–3F, which are plotted in logarithmic scale to visualize the extent of the work function tailing. Region II shows a narrower distribution with a maximum at 4.9 eV (Figure 3F), whereas the maximum in the distribution in the region I is 4.7 eV (Figure 3E). The histogram associated with the large FoV map (Figure 3D) is a combination of the maps in regions I (Figure 3E) and II (Figure 3F), suggesting that the effect of the local topography on the LEWF value is relatively minor. Previous studies based on Kelvin probe microscopy reported estimated global WF values in a similar range to those observed in Figure 3A (Kim et al., 2014). Consequently, we propose that the spatially resolved LEWF maps with large FoV are representative of the surface electronic landscape operating in the solar cell.

A variance of 66 meV is estimated from fitting a Gaussian function to the distribution of LEWF in region II (dotted line in Figure 3F). Interestingly, this value is very close to the mean potential energy fluctuation depth (γ) estimated from photoluminescence spectra of the film and the EQE spectrum of the device (see Figure S3), which are very close to the values reported by other groups on films of identical composition (Gokmen et al., 2013). This observation suggests that the LEWF landscape in Figure 3C is a manifestation of the potential energy fluctuation linked to clustering of Cu_Zn_ and Zn_Cu_ antisites in the sub-micrometer length scale (Bourdais et al., 2016). A similar broadening can be seen around the maximum LEWF histogram of the region I (Figure 3E), with an additional tail down to 4.2 eV.

Figure 3G shows a snapshot of electron photoemission maps (FoV of 93.2 μm) in the range of *E−E*_f_ values of 0–21 eV, which can be seen as a video in the Supplemental Information (Video S1). Photoemission is detected in hotspots across the surface at *E−E*_f_ below 4.25 eV, becoming more widespread across the surface at values above 4.75 eV. “Dark” lines appearing in the photoemission background correspond to large crystallites observed in scanning electron micrograph (see bottom of Figures 2B, 2C, and S4A). The out-of-plane crystallites promote photoelectron emission in different trajectories and cannot be collected through the 150-μm contrast aperture of the detector.

Video S1. 93.2-μm Field of View Photoemission Maps Showing Regions with Different Electron Emission Energy Threshold, Related to Figure 3 Photoemission hotspots are associated with sub-micron Sn(S,Se) surface domains.

Close examination of the LEWF landscape shows that photoemission hotspots are mainly observed in regions of smooth topography (region I). Consequently, these hotspots can be linked to local contrast in a chemical environment, acting as carrier shunting path. Interestingly, EDX mapping (Figure S4) and Raman microscopy (Figure S5) do not provide any clear contrasts of local structure and chemical composition across the surface. These observations provide strong evidence that the electron shunting paths are connected to surface domains, which can only be detected by virtue of the short inelastic mean free path (∼1 nm) of low-energy electrons in photoemission spectroscopy and the sub-micron lateral resolution of the EF-PEEM system.

### Chemical Identity of the Shunting Pathway

Localized photoemission spectra of areas associated with low and high LEWF are illustrated in Figure 4A. LEWF values are extracted by fitting an error function to the secondary electron edge, whereas the bands observed closer to the primary electron edge are related to the local valence band spectrum. The spectra in Figure 4A show that areas of high LEWF in regions I and II have very similar spectra, whereas the photoemission hotspots exhibit a rather different pattern. Subtracting the incident photon energy (21.2 eV) and applying a Tougaard background correction allow a clearer visualization of the local spectral features of the high-LEWF areas as shown in Figure 4B. To rationalize the chemical nature of these spectral features, valence band density of states (DOS) were calculated by DFT employing HSE06 functional. Figure 4C shows the DOS calculated for a supercell with a composition of Cu_2_ZnSn(S_0.25_Se_0.75_)_4_, which was used to decrease computational costs. As shown in Figure S6, the calculated band gap was 1.13 eV, which is in good agreement with the experimental 1.18 eV value obtained from diffuse reflectance spectrum and EQE spectrum of devices (Tiwari et al., 2017b). The computed DFT spectrum reproduces the main features observed in the valence band spectrum, particularly close to *E*_F_, although it tends to somewhat underestimate the energy of the deeper bands. This could be related to the intrinsic limitations of the HSE functional or the fact that a slightly different composition was used to limit the computational costs. In any case, the calculated spectrum reproduces the Cu 3*d* (between −1 and −6 eV) and Zn 3*d* orbitals (−10 eV), which are key distinctive features. The strong correlation between experimental and calculated spectra provides clear evidence that regions with high LEWF are associated with the main CZTSSe phase.

**Figure 5:**
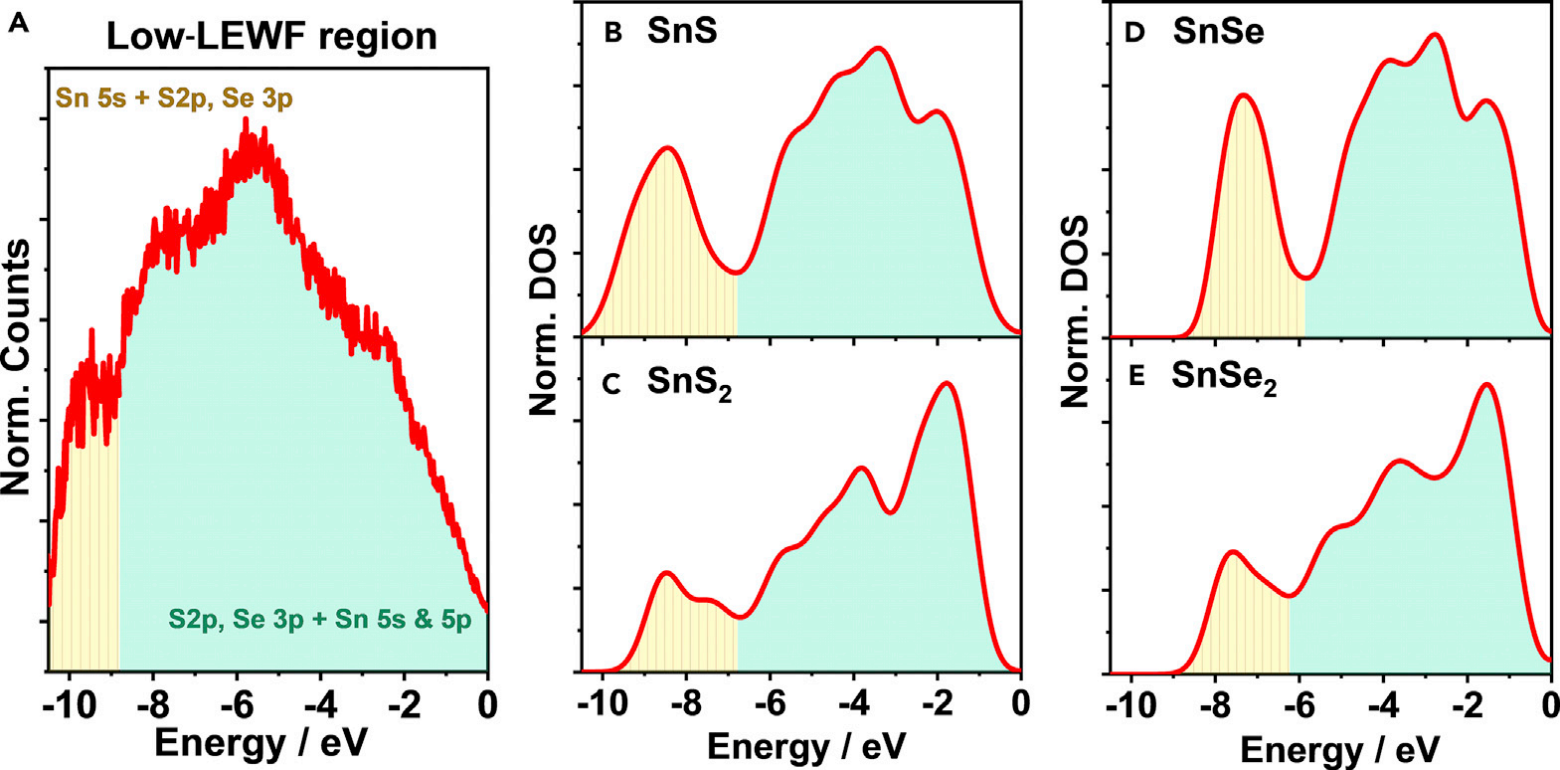
Valence Band Spectra of the Photoemission Hotspots (A–E) (A) Photoemission spectra from low-LEWF spots after subtracting the incident photon energy (21.2 eV) and applying a Tougaard background correction. Calculated DOS of the valence band of (B) SnS, (C) SnS_2_, (D) SnSe, and (E) SnSe_2_. DOS are calculated by DFT employing HSE06 functional and plotted with 0.4-eV Gaussian smearing to match the broadening in the experimental measurements. Elemental contributions to the valence and conduction band DOS are shown in Figures S7 and S8.

The valence band spectrum of low-LEWF segments exhibits different features when compared with the CZTSSe phase, as shown in Figure 5A. The significant attenuation of Cu 3*d* and Zn 3*d* bands strongly suggests that the LEWF hotspot is mainly composed of Sn chalcogenides. Figures 5B–5E contrast the calculated DOS for SnS, SnS_2_, SnSe, and SnSe_2_. Although slight differences can be seen in the position of the Sn 4*s* orbital for the S and Se phases, the line shapes are very similar and mostly determined by the Sn oxidation state. The broad spectral features of the 2*p* orbitals of the chalcogenide and the slightly sharper Sn 4*s* band suggest that the LEWF spots have a strong SnS and/or SnSe character (individual orbital contributions for the S and Se phases are shown in Figures S7 and S8). We have also calculated the DOS of ZnS and ZnSe (Figure S9), which are characterized by a very different line shape to the spectrum in Figure 5A. Furthermore, WF calculations of the SnS and SnSe 100 faces result in values between 4.24 and 4.31 eV (Figures S10 and S11), which are comparable to the photoemission hotspot features in our spectra. It should be mentioned that this value also matches experimental data (Ettema et al., 1992, Yao et al., 2017), although recent DFT studies employing more sophisticated functionals provided higher WF values (Burton and Walsh, 2013). Based on this analysis, it can be concluded that the most likely composition of the LEWF hotspots corresponds to Sn(S,Se) surface domains.

The formation of the sub-micron Sn(S,Se) surface domains are most probably generated as a result of partial SnS sublimation during the reactive annealing in the Se atmosphere. Indeed, it is well established that the CZTSSe crystallization occurs with a complex set of solid-gas phase reactions involving Sn(II) chalcogenides (Johnson et al., 2015, Davydova et al., 2018). EF-PEEM is inherently sensitive to the first few monolayers, therefore conventional diffraction and spectroscopic techniques would not be sensitive to this localized surface domain. Quantitative Rietveld refinement of XRD data on these films has concluded that no secondary phase of Sn(S,Se) is present above 1 wt% (Tiwari et al., 2017b).

## Discussion

**Figure 6:**
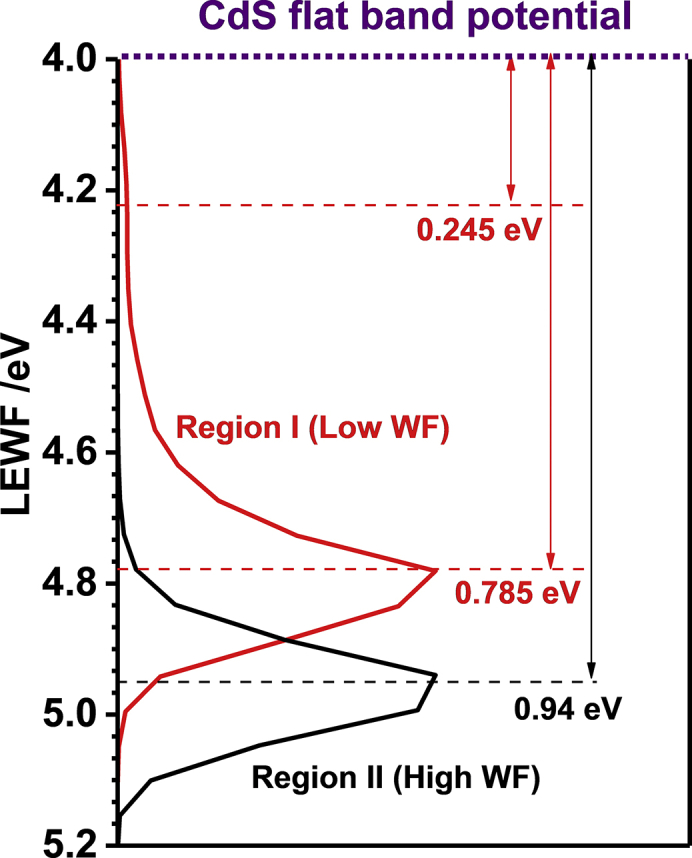
Comparison of the CdS Fermi Level and the LEWF Distributions in Regions I and II CdS Fermi level was obtained from impedance measurements (Figure S12). Ignoring significant changes in surface composition upon CdS deposition, contact potential differences larger than 0.8 V can be predicted from these measurements.

LEWF maps can also be used to rationalize the built-in potential at the CZTSSe/CdS heterojunction as illustrated in Figure 6. In principle, the built-in junction potential is determined by the difference in WF of both materials. The CdS WF was extracted from impedance measurements in contact with a redox-inactive electrolyte, as illustrated in Figure S12. We have used this approach given the vast amount of data in the literature that allows us to validate our assumptions, also in view of the strong dependence of this parameter on preparation and post-treatment protocols (Ginley and Butler, 1978, Meissner et al., 1988). Interestingly, our representation in Figure 6 suggests that the conduction band offset between CZTSSe and CdS is between 0.3 and 0.4 eV, which falls within the range reported in previous works (Sardashti et al., 2015, Terada et al., 2015, Crovetto and Hansen, 2017). We could also estimate a difference of 0.8 eV between the CdS Fermi level and the maximum in the LEWF distribution of the “pure” CZTSSe phase. In region I, this difference is approximately 0.7 eV, decreasing even further to 0.2 eV in the low-LEWF hotspot domains. It should be mentioned that these arguments do not consider elemental mixing during the deposition of CdS, in particular Zn and Cd, which has been recently shown by Bär et al.(Bär et al., 2017) Elemental mixing will have an effect on the overall interfacial potential, which in turn is dependent on the film preparation and post-treatment protocols. In any case, the conditions in which CdS is conventionally deposited are unlikely to affect the chemical integrity of interfacial Sn(S,Se) phases (Ortega-Borges, 1993, Bar et al., 2011, Di Mare et al., 2016, Bär et al., 2017). Consequently, surface Sn(S,Se) domains are expected to act as shunting paths, decreasing the built-in potential the CZTSSe/CdS junction.

The complex electronic landscape unveiled by our photoemission microscopy studies shed new light into the origin of the voltage losses in CZTSSe devices, which is commonly linked to band gap fluctuations and/or electrostatic potential fluctuation (Rey et al., 2017). These electronic fluctuations, mainly connected to Cu-Zn disorder, are commonly described either in terms of a characteristic amplitude (γ) or the so-called Urbach tails (*E*_U_). In our film and devices, low-temperature photoluminescence (PL) and EQE measurements provide γ and *E*_U_ values of 68 and 45 meV, respectively (Figure S3). Experimental studies have shown a correlation between *E*_U_ (or γ) and *V*_OC_ deficiency in CZTSSe devices with power conversion efficiency ranging from 6% to 11%, but this trend falls outside those observed for GaAs, c-Si, CIGSe, and a-Si (Gokmen et al., 2013, Bourdais et al., 2016),(Miller et al., 2014). On the other hand, surface Sn(S,Se) domains can play a key role in the voltage losses of the devices through their characteristic low LEWF. Shunting via surface Sn(II) domains appears consistent with a recent study showing improvement in cell performance by air oxidation and exposure to ammonium hydroxide (Sardashti et al., 2016), as well as ammonium sulfide etching (Xie et al., 2014). Multiple Sn oxidation states may also play a substantial role in power conversion losses in other absorbers such as Cu_2_SnS_3_ (Baranowski et al., 2014, Tiwari et al., 2017a), SnS (Sinsermsuksakul et al., 2014), and Sn-based organohalide perovskites (Noel et al., 2014). Our observations are also consistent with a growing number of reports pointing toward Sn disorder, rather than Cu/Zn, as the key structural and electronic defects limiting the efficiency of CZTSSe devices (Kattan et al., 2015, Kattan et al., 2016, Wallace et al., 2017, Park et al., 2018).

### Conclusions

High-resolution EF-PEEM uncovered a highly complex surface electronic landscape of CZTSSe films, providing a new insight into the voltage losses in PV devices. Photoemission maps show large portions of the surface with LEWF values between 4.7 and 4.9 eV and valence band spectra characteristic of CZTSSe as rationalized by DFT. These measurements also reveal sub-micron electron emission hotspots with LEWF as low as 4.2 eV. Local photoemission spectral analysis suggests that the chemical composition of the hotspots is dominated by Sn(S,Se). Although different local CZTSSe phases, as well as other secondary phases (e.g. ZnS, ZnSe) can be present in these complex films, which are difficult to detect by conventional diffraction or spectroscopy techniques, our EF-PEEM data clearly show that their contributions to the photoemission landscape is relatively minor when compared with surface Sn(S,Se) domains. Our analysis also predicts that pure CZTSSe domains can promote built-in potentials as high as 0.8 V and PV performances comparable to those of CIGSe solar cells. However, these Sn(II) states can act as shunt pathways, strongly decreasing the device voltage. Finally, the outstanding energy and spatial resolution of EF-PEEM offers a unique view of the surface electronic landscape of these complex materials, not only visualizing the distribution of carrier shunting paths but also uncovering their chemical identity.

### Limitations of Study

One of the interesting outcomes of our studies is the correlation between the spatial distribution of LEWF in *films* and the depth of band tails estimated from *device* measurements. However, such a correlation must be confirmed by investigating a large number of films and cells. The effect of CdS deposition on the electronic landscape of CZTSSe is another important aspect that requires further assessment. The chemical nature of the photoemission hotspots should be further scrutinized by surface-sensitive microscopic techniques such as X-ray photoelectron emission microscopy. Finally, more sophisticated and computationally expensive DFT calculations employing spatially restricted cells (rather than periodic boundaries) could provide a more accurate description of the local valence band spectra of surface-confined domains.

## Methods

All methods can be found in the accompanying Transparent Methods supplemental file.

## Data and Software Availability

The data in this paper is freely available from the University of Bristol Data Repository at https://doi.org/10.5523/bris.3ms4y4q5ois072dq5vtfbtqnrt.

## Supplemental Information

Document S1. Transparent Methods, Figures S1–S12, and Table S1

## Footnotes

Supplemental Information includes Transparent Methods, 12 figures, and 1 table and can be found with this article online at https://doi.org/10.1016/j.isci.2018.10.004.
