# Supplementary material for: Mapping Shunting Paths at the Surface of Cu2ZnSn(S,Se)4 Films via Energy-Filtered Photoemission Microscopy
Source: iScience. 2018 Oct 13;9:36–46. doi: 10.1016/j.isci.2018.10.004 (PMC6215027; doi:10.1016/j.isci.2018.10.004)
Supplement: Document S1. Transparent Methods, Figures S1–S12, and Table S1 [file mmc1.pdf]

ISCI, Volume *9*

Supplemental Information

Mapping Shunting Paths at the Surface of $Cu_2ZnSn(S,Se)_4$ Films via Energy-Filtered Photoemission Microscopy

Devendra Tiwari, Mattia Cattelan, Robert L. Harniman, Andrei Sarua, Ali Abbas, Jake W. Bowers, Neil A. Fox, and David J. Fermin

Supplemental Information

S1. Transparent methods

Figure S1 Conducting atomic force micrographs for regions I and II

Figure S2 XPS spectra of CZTSSe film before and after Ar plasma treatment

Table S1 Metal ratio estimated from XPS before and after plasma treatment.

Figure S3 Electrostatic potential fluctuations amplitude (γ) and Urbach tail energy (E_u) analysis

Figure S4 Scanning electron micrograph and EDX elemental mapping

Figure S5 Raman spectroscopy and microscopy of CZTSSe film

Figure S6 Partial density of states $Cu_2ZnSn(S_{0.25}Se_{0.75})_4$

Figure S7 Partial density of states of SnS and SnS_2

Figure S8 Partial density of states of SnSe and $SnSe_2$

Figure S9 Partial density of states of ZnS and ZnSe

Figure S10 Work function estimation of the SnS 100 face

Figure S11 Work function estimation of the SnSe 100 face

Figure S12 Mott-Schottky plot of CdS CBD film on FTO.

S2. Supplemental references

S1. Transparent methods

S1.1 Molecular precursor solutions. CZTSSe films and devices are prepared using an earlier reported protocol.(Tiwari *et al.*, 2016, 2017) This involves spin-coating onto a 500 nm Mo coated glass a single precursor solution containing $CuCl_2.2H_2O$, $ZnCl_2$ and $SnCl_2.2H_2O$, as well as thiourea in N-Dimethyl formamide (DMF) – 2-Propanol mixtures. The concentrations of each component were $CuCl_2.2H_2O$: 0.41 M, $ZnCl_2$: 0.27 M, $SnCl_2.2H_2O$: 0.25 M, and thiourea: 0.95 M. While DMF provide good solubility of the various metal salts and thiourea, 2-Propanol ensures a good wettability on the Mo substrate.

S1.2 Film thermolysis and etching. The films are annealed under Se atmosphere at 550 ^{o}C for 30 minutes. The Cu/(Zn+Sn), Zn/Sn and Se/S ratios estimated from EDX analysis and quantitative XRD were 0.82, 1.11 and 2.8, respectively. The metal ratio in the films closely match those in the precursor solution. The films are etched in 10 % aqueous KCN solution for 30 seconds prior to any characterization study or device completion.

S1.3 Device competition and characterization. Devices are completed by depositing a 60 nm CdS layer using established chemical bath deposition,(Tiwari *et al.*, 2017) followed by deposition of i-ZnO and Al: ZnO as window and transparent conducting oxide (TCO) using RF-sputtering. No blocking layer, preventing Na diffusion from glass substrate, were used. Measurements were done by directly connecting the (TCO), without metallic contact or anti-reflective coating, on mechanically scribed squares of 0.25 cm^2. The device performance is analyzed at 296 K using AM 1.5G AAA class solar simulator (Wacom, Japan), with a power density of 100 mW cm^{-2} as measured against a calibrated reference cell from Newport. The measurements are performed by placing the sample in the center of the illumination area. The illumination divergence over such small area of cell with an area of 0.25 cm^2 is considered to be negligible. Statistical analysis of 20 devices showed variations of less than 15% from the mean value of key PV indicators.(Tiwari *et al.*, 2017) Temperature variation of the open-circuit voltage is measured using a liquid nitrogen flow cryostat (Linkam) and Solartron Modulab for data acquisition.

S1.4 Microscopy and spectroscopy mapping. High resolution scanning transmission electron microscopy (HR-STEM) is performed using a FEI Tecnai F20 (S)TEM also equipped with silicon drift detector (SDD) for elemental mapping with energy dispersive analysis of X-rays (EDX). Film morphology is analyzed using SEM (Jeol iT300) in secondary electron detection mode. Elemental mapping is recorded using energy dispersive analysis of X-rays (EDX) with an Oxford X-max 80 mm^2 detector. Film topography and surface conductance are studied with a tunneling atomic force microscopy (TUNA Bruker multimode VIII with Nanoscope V controller and PeakForce feedback) using Pt/Ir coated tip with nominal radius of 20 nm and applying 500 mV bias. Raman Spectroscopy measurements are performed using a 532-nm excitation from a diode laser and a Renishaw In-Via spectrometer.

S1.5 Energy-filtered photoemission spectroscopy (PES) and microscopy (PEEM). EF-PES and EF-PEEM are performed in a ScientaOmicron system with XPS and PEEM chambers, with a base pressure of 4×10^{-11} mbar. For sample preparation, 0.5 kV Ar plasma etching is performed at room temperature at 45 ^{o} (base pressure: 5 x 10^{-5} mbar) for 5 minutes. Room temperature XPS spectra are acquired with monochromatic Al Kα source before and after plasma etching of the film. As shown in the supplemental information (**Figure s2** and **table s1**), Ar plasma etching reduces C and O 1s peaks to negligible background levels while the Cu/Zn/Sn ratio remains unaffected. This was achieved by careful optimization of the power, time and angle the Ar plama cleaning. The quantification of metal ratio is done using Cu 3*p* at 77 eV, of Zn 3*p* at 90 eV and of

Sn 3*d* at 494 eV, as these photoemission lines are not significantly affected by changes in C and O content (**table s1**).

Following the XPS analysis, samples are transferred to the EF-PEEM chamber under UHV conditions. He I (21.2 eV) light source is used as excitation source. EF-PEEM in NanoESCA II combines a conventional electrostatic PEEM with an aberration corrected energy filter. All measurements were carried out at room temperature with 50 eV pass energy and 140 meV overall energy resolution, as calculated by fitting the Fermi edge of a clean metallic substrate at the same measurement conditions. The photoelectron energy is measured with respect to the Fermi level (E_F) of the sample holder. Thus, the effective WF is the value of $E-E_F$ for the photoemission threshold.(He *et al.*, 2016) LEWF maps, recorded with a contrast aperture of 150 µm over two different field-of-views: 93.2 µm and 14.6 µm, are obtained by fitting the 600 × 600 camera pixels spectra with an error function. Local valence band spectra are recorded using a mechanical aperture, while the contrast aperture is fully opened to avoid limiting k-space sampling. The spectra (as shown in **Figure 4a**) are measured over regions of 1.5 µm, 6.5 µm and 12 µm for low LEWF Region I, high LEWF Region I and high LEWF Region II, respectively.

S1.6 First-principles calculations. Density of states are calculated using CASTEP DFT pseudopotential planewave calculation package.(Payne *et al.*, 1992; Clark *et al.*, 2005) A 96-atom supercell with the composition $Cu_2ZnSn(S_{0.25}Se_{0.75})_4$ is used, which closely represent the S/Se ratio. While for the binary compositions i.e. SnS, SnS_2, SnSe, $SnSe_2$, ZnS and ZnSe, their respective unit cells are used as the starting point. Geometry optimizations are performed using PBESOL functional for all but the pseudo-layered structures: SnS_2 and $SnSe_2$ for which PBE functional with Grimme dispersion correction(Grimme, 2006) (accounting for the van der Waals interactions) is employed. Norm-conserving on-the-fly pseudopotentials with energy cutoff of 1350 eV are implemented and Brillouin zone is sampled with 0.015 $Å^{-1}$ spaced Monkhorst-Pack grid. The structures are relaxed with strict tolerances of $1x10^{-7}$ eV/atom for energy, 1 x 10^{-3} eV/Å for force and 1 x 10^{-6} Å for displacement. The lattice parameters for all the optimized structures lie within 0.6 % of the experimentally known values. For density of states (DOS) calculations, the functional is switched to HSE06 hybrid. Given that all the composition but SnS_2, $SnSe_2$ have rigid 3-dimensional structure (cubic, tetragonal orthorhombic), it is assumed that the layer dependence of DOS should be negligible. Even in case of SnS_2 and $SnSe_2$ with trigonal pseudo-layered structure, it has been shown the layer dependence or size confinement neither affect relative band positions nor the nature of band gap.(Gonzalez and Oleynik, 2016) Thus, it is assumed here the DOS calculated from the bulk structures should provide qualitatively reasonable picture when assigning bands of a room-temperature measured valence band spectrum.

S1.7 CdS flat-band potential analysis. Space-charge capacitance of CdS films are calculated from impedance spectroscopy in a contact with an aqueous electrolyte at neutral pH (Na_2SO_3), employing Ag/AgCl and Pt as reference and counter electrodes, respectively. Impedance spectra were recorded with a Solatron Modulab system between 11 Hz and 9.9 kHz using 11 mV rms amplitude. The response was fitted using an RC circuit, showing negligible frequency dependence of the capacitance value.

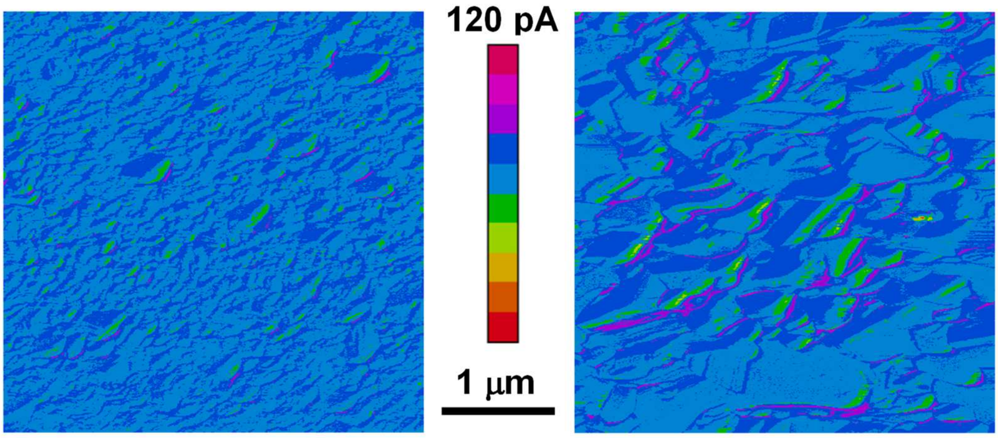

Figure S1 (related to Figure 2f,g): Conducting atomic force micrographs for regions I (left) and II (right) of CZTSSe films. The corresponding topographic images are included in Figure 2 of the main manuscript.

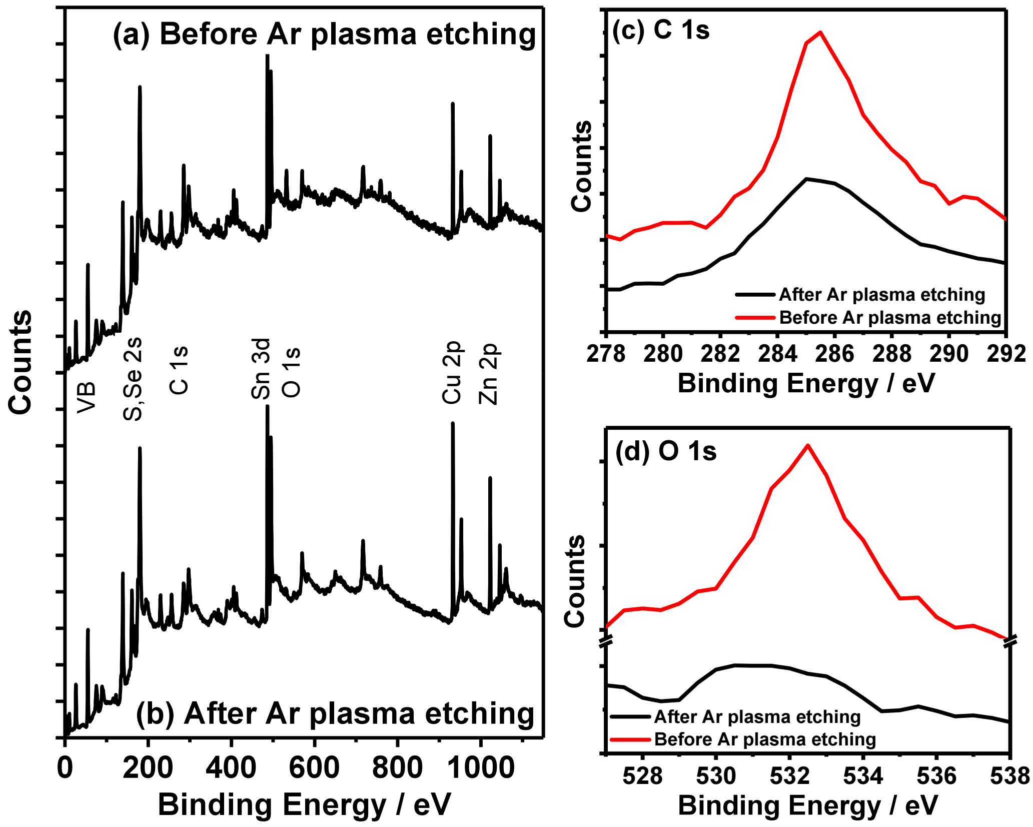

Figure S2 (related to Figure 3): X-ray photoelectron spectra (XPS) of CZTSSe film before (a) and after (b) a 0.5 kV Ar plasma treatment for 5 minutes. The spectra show considerable lowering of C and O s peaks (c) and (d), though metal ratios remain same within the detection limit.

Table S1 (related to Figure 3). Metal ratio estimated from XPS before and after plasma treatment.

	[Cu]/([Zn]+[Sn])	[Zn]/[Sn]
**After**	0.76	1.6
**Before**	0.75	1.58

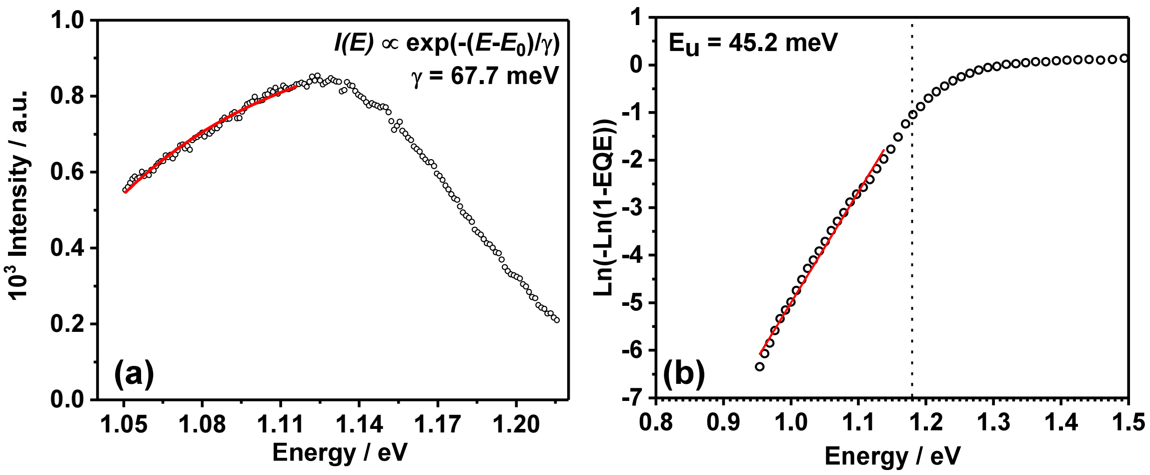

Figure S3 (related to Figure 4f): (a) Electrostatic potential fluctuations amplitude (γ) estimated from fitting the low energy region of a photoluminescence spectrum collected at 6K. The spectral response was fitted to an exponential decay of density of states at the band edges i.e. band tail behavior (equation shown as inset). (b) Urbach tail energy (E_u) extracted from the sub-bandgap absorption region in external quantum efficiency spectrum (EQE) of CZTSSe solar cell.

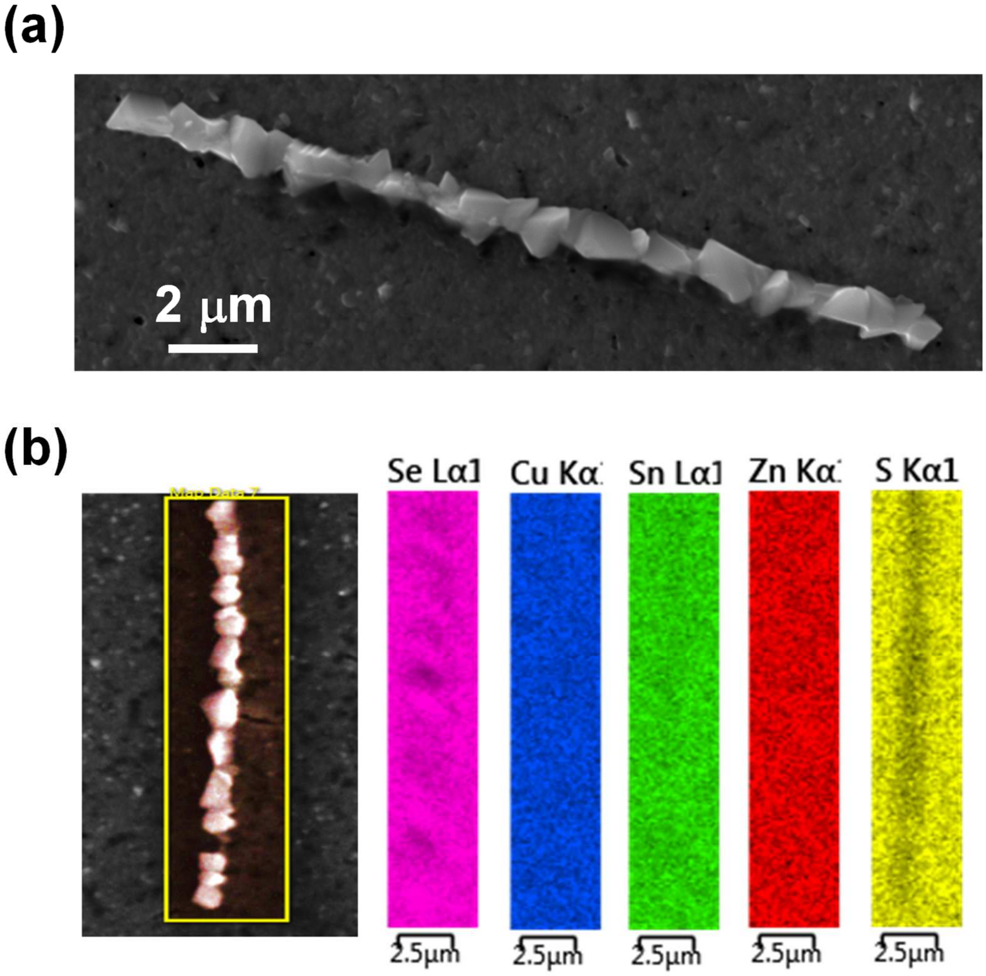

Figure S4 (related to Figure 3): Scanning electron micrograph (a) and EDX elemental mapping (b), of the linear features at the surface of the CZTSSe films. EDX analysis is performed at 20 kV and low probe current to enhance surface specificity. Within the instrumental capabilities, these features show similar composition to bulk CZTSSe. Large grains appear slightly Se rich.

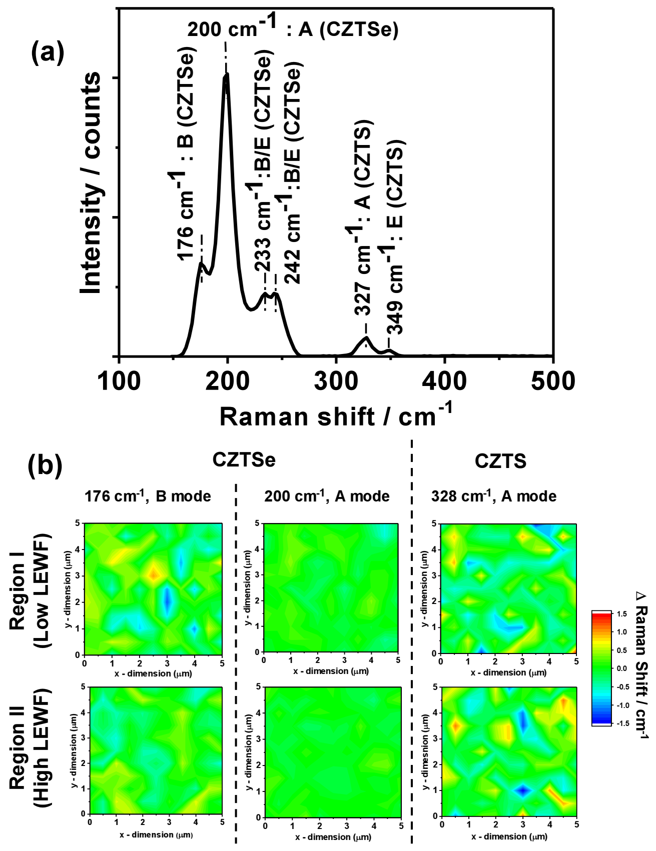

Figure S5 (related to Figure 3): A representative Raman spectrum of CZTSSe film under 532 nm excitation (a) and Raman maps displaying the changes in position of the three major peaks in Raman spectrum of CZTSSe across a 5 x 5 μm^2 area in pure CZTSSe region and region infested with surface impurity (b). The spectral resolution of the measurements is 1.5 cm^{-1}. Figure S5b reveals remarkably little contrast in the selected regions. A representative spectrum of the entire film is shown in Figure S5a, featuring sharp peaks associated with the A mode of the sulfur (328 cm^{-1}) as well as the A (200 cm^{-1}) and B (176 cm^{-1}) modes of the selenide forms of the kesterite lattice. The spectrum does not show any binary or ternary chalcogenide phases that can be detected with 532 nm excitation. The A and B mode peaks are strongly dependent on the S/Se ratio, therefore contrast in the Raman shifts maps can provide information on spatial fluctuations of chalcogen composition. However, the maps across regions I and II show only slight shifts within the spectral resolution of the measurement (1.5 cm^{-1} from the mean value). Considering the large capture cross section of the CZTSSe film (band gap 1.18 eV), the penetration depth of the laser excitation is expected to be below 100 nm.

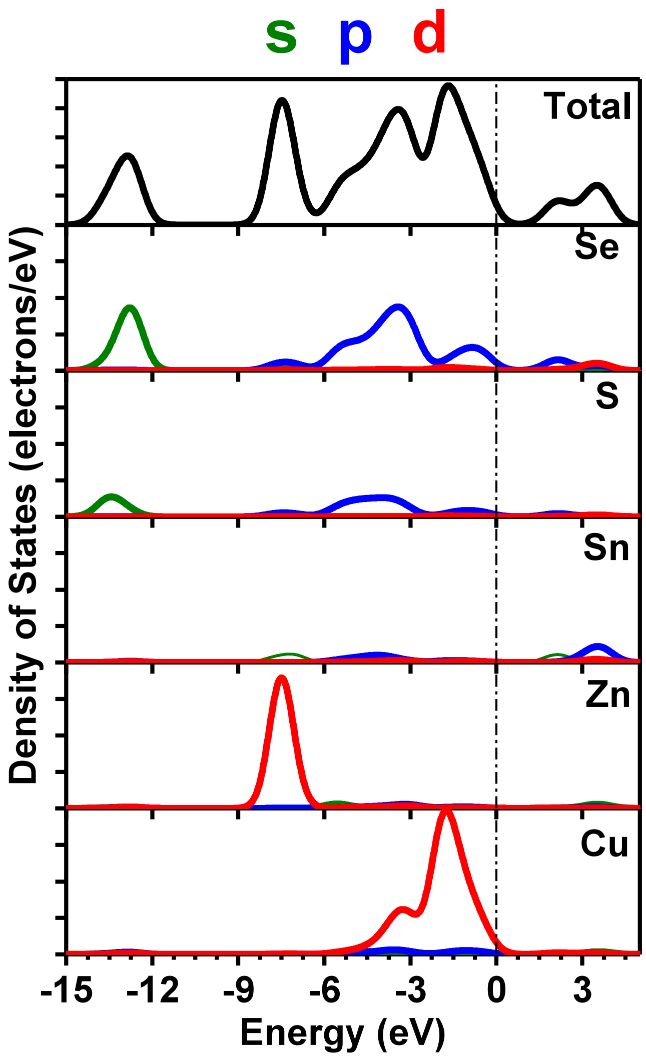

Figure S6 (related to Figure 4): Partial density of states $Cu_2ZnSn(S_{0.25}Se_{0.75})_4$ calculated by DFT employing HSE functionals. Orbital contributions are shown from the various elements (0.4 eV gaussian smearing).

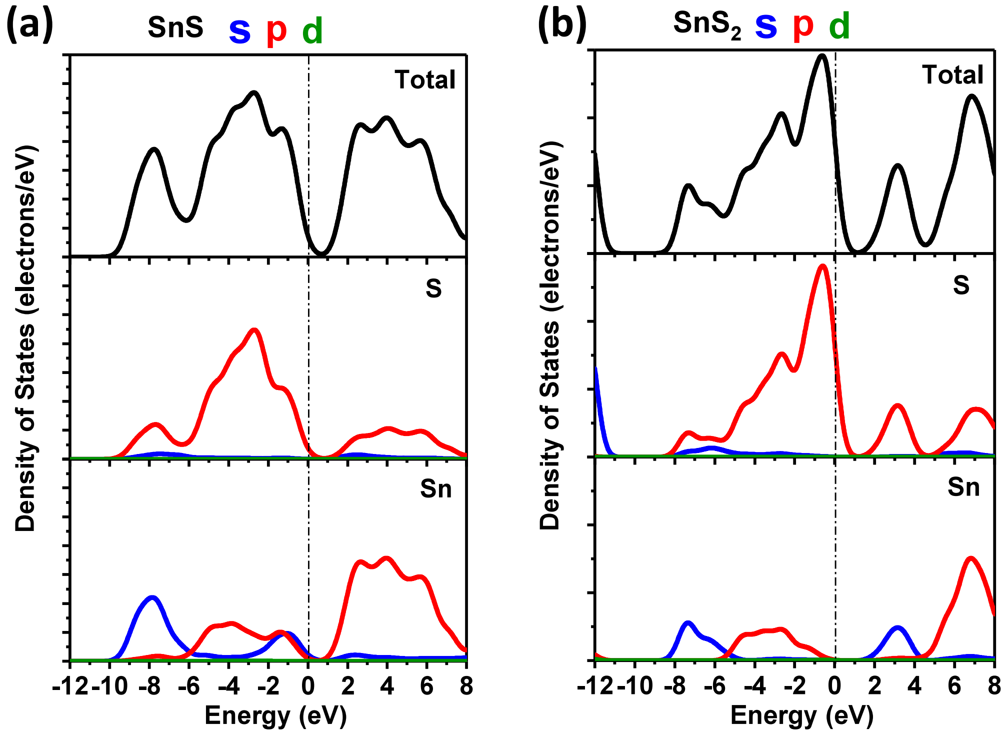

Figure S7 (related to Figure 4 and 5): Partial density of states of SnS (a) and SnS_2 (b) plotted with 0.4 eV Gaussian smearing.

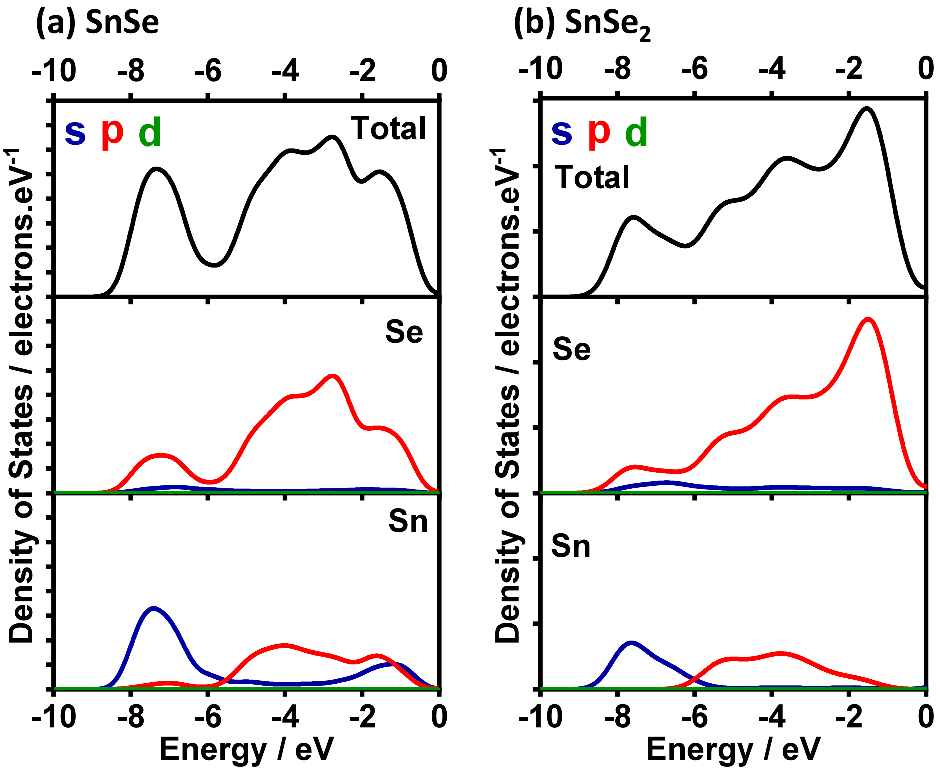

Figure S8 (related to Figure 4 and 5): Partial density of states of SnS (a) and SnS_2 (b) plotted with 0.4 eV Gaussian smearing.

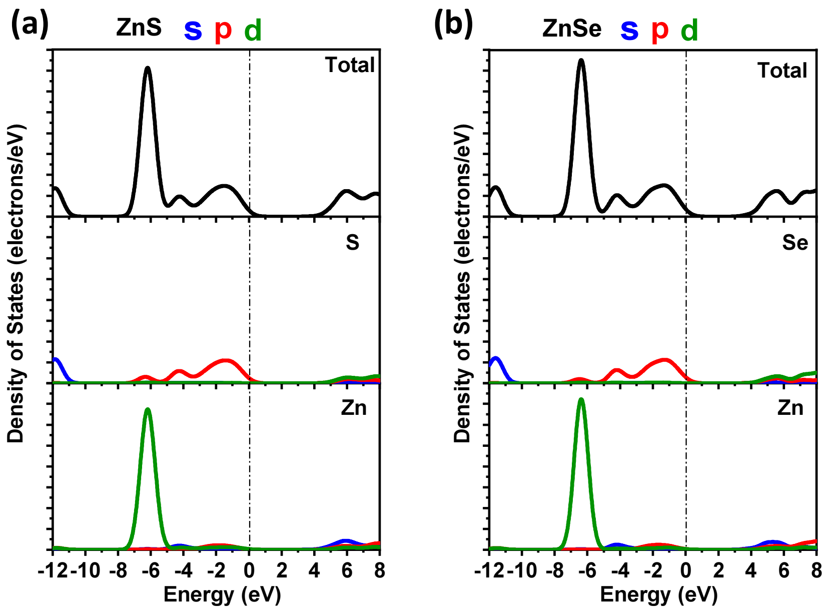

Figure S9 (related to Figure 4 and 5): Partial density of states of ZnS (a) and ZnSe (b) plotted with 0.4 eV Gaussian smearing.

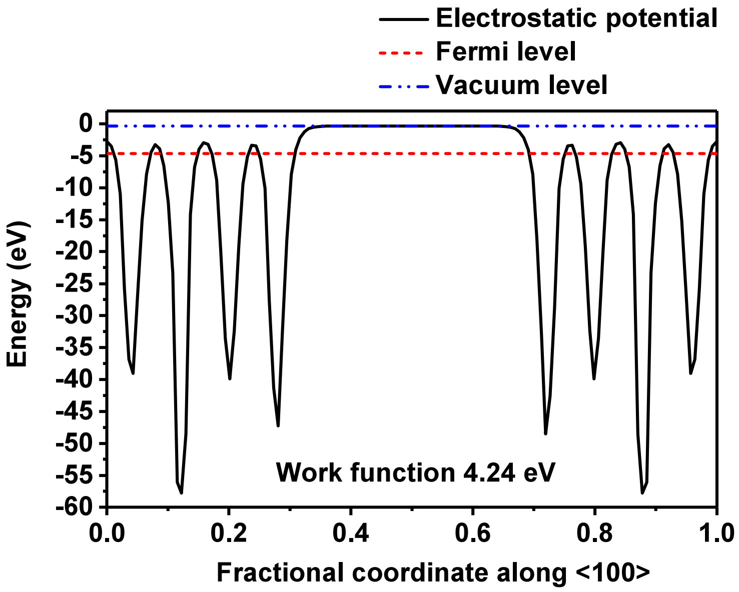

Figure S10 (related to Figure 4 and 5): Work function estimation of the SnS 100 face.

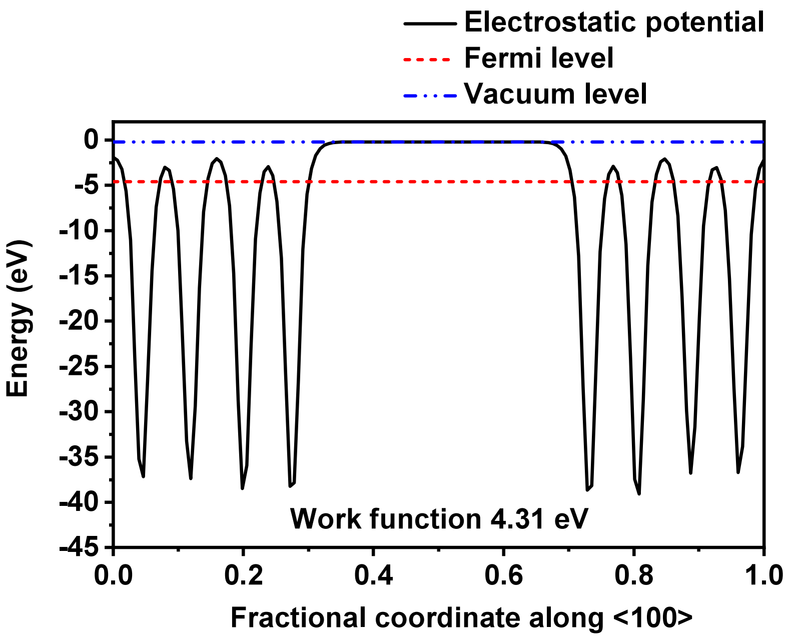

Figure S11 (related to Figure 4 and 5): Work function estimation of the SnSe 100 face.

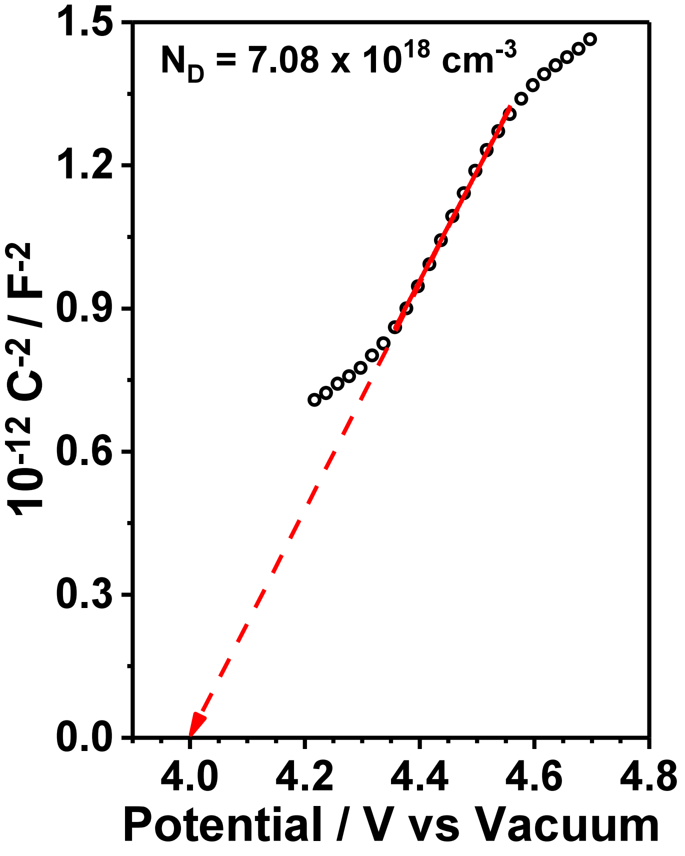

Figure S12 (related to Figure 6): Mott-Schottky plot of CdS film on FTO in 0.1 M sodium sulfite electrolyte. CdS films are deposited using the same conditions as for CZTSSe solar cells.

S2. Supplemental references

Clark, S. J. *et al.* (2005) 'First principles methods using CASTEP', *Zeitschrift fur Kristallographie*, 220, pp. 567–570. doi: 10.1524/zkri.220.5.567.65075.

Gonzalez, J. M. and Oleynik, I. I. (2016) 'Layer-dependent properties of SnS2 and SnSe2 two-dimensional materials', *Physical Review B*, 94, pp. 125443-1–10. doi: 10.1103/PhysRevB.94.125443.

Grimme, S. (2006) 'Semiempirical GGA-Type Density Functional Constructed with a Long-Range Dispersion Correction', *Journal of computational chemistry*, 27, pp. 1787–1799. doi: 10.1002/jcc.

He, Y. *et al.* (2016) 'High resolution angle resolved photoemission with tabletop 11 eV laser', *Review of Scientific Instruments*, 87, pp. 011301-1–11. doi: 10.1063/1.4939759.

Payne, M. C. *et al.* (1992) 'Iterative minimization techniques for ab initio total-energy calculations: Molecular dynamics and conjugate gradients', *Reviews of Modern Physics*, 64, pp. 1045–1097. doi: 10.1103/RevModPhys.64.1045.

Tiwari, D. *et al.* (2016) 'Cu_2ZnSnS_4 thin-films generated from a single solution based precursor: the effect of Na and Sb doping', *Chemistry of Materials*, 28, pp. 4991–4997. doi: 10.1021/acs.chemmater.6b01499.

Tiwari, D. *et al.* (2017) 'Spectroscopic and Electrical Signatures of Acceptor States in Solution Processed $Cu_2ZnSn(S,Se)_4$ Solar Cells', *J. Mater. Chem. C*, 5, pp. 12720–12727. doi: 10.1039/C7TC03953K.
